# Metabolomic profiles are reflective of hypoxia-induced insulin resistance during exercise in healthy young adult males

**DOI:** 10.1152/ajpregu.00076.2021

**Published:** 2021-05-05

**Authors:** Lee M. Margolis, J. Philip Karl, Marques A. Wilson, Julie L. Coleman, Arny A. Ferrando, Andrew J. Young, Stefan M. Pasiakos

**Affiliations:** ^1^United States Army Research Institute of Environmental Medicine, Natick, Massachusetts; ^2^Oak Ridge Institute of Science and Education, Oak Ridge, Tennessee; ^3^Department of Geriatrics, Center for Translational Research in Aging and Longevity, Donald W. Reynolds Institute on Aging, University of Arkansas for Medical Sciences, Little Rock, Arkansas; ^4^Henry Jackson Foundation for the Advancement of Military Medicine, Bethesda, Maryland

**Keywords:** branched-chain amino acids, fatty acids, glycogenolysis, high altitude, substrate oxidation

## Abstract

Hypoxia-induced insulin resistance appears to suppress exogenous glucose oxidation during metabolically matched aerobic exercise during acute (<8 h) high-altitude (HA) exposure. However, a better understanding of this metabolic dysregulation is needed to identify interventions to mitigate these effects. The objective of this study was to determine if differences in metabolomic profiles during exercise at sea level (SL) and HA are reflective of hypoxia-induced insulin resistance. Native lowlanders (*n* = 8 males) consumed 145 g (1.8 g/min) of glucose while performing 80-min of metabolically matched treadmill exercise at SL (757 mmHg) and HA (460 mmHg) after 5-h exposure. Exogenous glucose oxidation and glucose turnover were determined using indirect calorimetry and dual tracer technique ([^13^C]glucose and [6,6-^2^H_2_]glucose). Metabolite profiles were analyzed in serum as change (Δ), calculated by subtracting postprandial/exercised state SL (ΔSL) and HA (ΔHA) from fasted, rested conditions at SL. Compared with SL, exogenous glucose oxidation, glucose rate of disappearance, and glucose metabolic clearance rate (MCR) were lower (*P* < 0.05) during exercise at HA. One hundred and eighteen metabolites differed between ΔSL and ΔHA (*P* < 0.05, *Q *<* *0.10). Differences in metabolites indicated increased glycolysis, tricarboxylic acid cycle, amino acid catabolism, oxidative stress, and fatty acid storage, and decreased fatty acid mobilization for ΔHA. Branched-chain amino acids and oxidative stress metabolites, Δ3-methyl-2-oxobutyrate (*r* = −0.738) and Δγ-glutamylalanine (*r* = −0.810), were inversely associated (*P* < 0.05) with Δexogenous glucose oxidation. Δ3-Hydroxyisobutyrate (*r* = −0.762) and Δ2-hydroxybutyrate/2-hydroxyisobutyrate (*r* = −0.738) were inversely associated (*P* < 0.05) with glucose MCR. Coupling global metabolomics and glucose kinetic data suggest that the underlying cause for diminished exogenous glucose oxidative capacity during aerobic exercise is acute hypoxia-mediated peripheral insulin resistance.

## INTRODUCTION

Acute high-altitude (HA) exposure (<8 h) suppresses exogenous glucose oxidation during steady-state aerobic exercise compared with metabolically matched exercise at sea level (SL) (1–4). In two separate studies (1, 4), our laboratory observed that consuming exogenous glucose during metabolically matched, steady-state aerobic exercise was associated with higher concentrations of circulating glucose and insulin, and lower rates of exogenous glucose oxidation during acute HA exposure compared with SL. Furthermore, glucose rate of disappearance (*R*_d_) and metabolic clearance rate (MCR), kinetic measures indicative of glucose uptake and utilization (5, 6), were both lower at HA compared with SL (1). Collectively, these metabolic dysregulations suggest that acute hypoxia elicits peripheral insulin resistance in healthy exercising adults (7). Although our glucose kinetics and circulating glucose and insulin responses suggest impaired insulin sensitivity as a potential mechanism contributing to the reduction in exogenous glucose oxidation, a better understanding of this metabolic dysregulation is required to identify interventions to mitigate these effects of acute HA exposure.

Nontargeted metabolomics analysis is a methodological approach that may provide greater insight into metabolic alterations that manifest when exogenous glucose oxidation is impaired at HA. This approach provides a comprehensive and sensitive analysis, allowing for the simultaneous measurement of hundreds to thousands of metabolites which can capture changes in whole body metabolism (8, 9). Metabolite profiling may provide greater insight into hypoxia-induced insulin resistance as a mechanism resulting in changes in metabolic pathways associated with impaired substrate oxidation, mobilization, and storage. Recent investigations have used metabolite profiling to gain a greater understanding into metabolic alterations with altitude acclimatization of ≥14 days (10, 11). This approach has also been effectively used to characterize metabolic dysregulation between healthy individuals and those with insulin resistance or type 2 diabetes (12–14). In addition, individual metabolites of branched-chain amino acid (BCAA) metabolism and oxidative stress have been identified as markers of these disease states (12–14). Identifying similar patterns in healthy adults during aerobic exercise at HA would strengthen evidence of hypoxia-induced insulin resistance and aid in identifying potential therapeutic targets.

The objective of this study was to assess the effects of acute HA exposure on changes in global metabolomic profiles during aerobic exercise while consuming carbohydrate. We hypothesized that metabolomic profiles would differ between SL and HA, and that these differences would be reflective of hypoxia-induced insulin resistance. In addition, metabolites that differ between SL and HA will be associated with reductions in exogenous glucose oxidation rate, glucose *R*_d_, and MCR at HA compared with SL.

## METHODS AND MATERIALS

### Participants

Participants in this study were a part of a larger randomized crossover study that examined the impact of acute HA (hyperbaric hypoxia) on exogenous glucose oxidation and glucose turnover during metabolically matched, steady-state aerobic exercise compared with SL (1). Eight healthy, recreationally active men (age: 23 ± 2 yr) completed the study. Individuals were excluded from participation if they had any metabolic, cardiovascular, or gastrointestinal disorders, prior diagnosis of high-altitude pulmonary edema or high-altitude cerebral edema, evidence of apnea or other sleeping disorders, presence of asthma or respiratory tract infection (<1 mo of data collection), taking medications that interferes with oxygen delivery, anemia (HCT < 38% and Hgb < 12.5 g/dL), sickle cell anemia/trait, born at altitudes >2,100 m, living at altitudes >1,200 m, were smokers, refused to abstain from alcohol, smokeless tobacco, and dietary supplement use during the study, had musculoskeletal injury that compromised ability to exercise, or donated blood with 8 wk of beginning the study. All data collection took place at the United States Army Research Institute of Environmental Medicine (USARIEM, Natick, MA), during November and December 2018. This study was approved by the Institutional Review Board at the US Army Medical Research and Development Command (MRDC, Fort Detrick, MD; www.clinicaltrials.gov; NCT03851744).

Height (Seritex, Inc., Carlstadt, NJ), body mass (WB-110A, Tanita, Tokyo, Japan), and body composition (dual energy X-ray absorptiometry, DPX-IQ, GE Lunar Corporation, Madison, WI), were used to characterize the participants (body mass: 83 ± 9 kg, height: 178 ± 7 cm, body mass index: 26 ± 3 kg/m^2^, fat mass: 21 ± 5 kg, fat-free mass: 63 ± 5 kg). Peak oxygen uptake (V̇o_2peak_) was assessed during a progressive intensity, treadmill (Trackmaster TMX425C, Newton, KA) running exercise test using an indirect, open circuit respiratory system (True Max 2400, Parvo Medics, Sandy, UT) to prescribe exercise intensities. Participants completed assessments of V̇o_2peak_ under SL (4.3 ± 0.2 L/min) and HA (2.9 ± 0.2 L/min) conditions.

### Study Design

As previously described (1), to normalize the effects of diet and exercise on endogenous carbohydrate stores before the experiments, 48 h before testing, participants completed a glycogen depletion protocol by cycling (Lode, BV, The Netherlands) at various intensities until failure (15), and were then fed a controlled diet (5.9 ± 0.2 g/kg/day carbohydrate, 1.2 ± 0.1 g/kg/day protein, and 1.0 ± 0.1 g/kg/day fat) before each study arm. At the conclusion of the normalization phase, participants reported to the hypobaric chamber after a 10-h overnight fast to complete the trial under SL (757 mmHg) and HA (460 mmHg) conditions. To match our previous work (16), participants rested quietly while exposed to SL or HA conditions for 5 h, and then they completed 80 min of metabolically matched, steady-state exercise on a treadmill, while consuming 145 g of glucose (1.8 g/min). Glucose turnover was assessed using 6-6-[^2^H_2_]glucose tracer methodologies. Indirect calorimetry and breath sampling for ^13^C/^12^C expired in CO_2_ were used to determine carbohydrate and fat oxidation during exercise at SL and HA. Blood samples for metabolomics analysis were collected 20 min before exercise under resting, fasted conditions at SL, and after 40 min of aerobic exercise at both SL and HA conditions. After a minimum 7-day washout period, participants returned to the laboratory to complete the second arm of the investigation. Treatment (SL vs. HA) order was randomized using a random numbers generator to avoid order bias.

### Steady-State Treadmill Exercise

After 48 h of controlled feeding and exercise, participants reported to the hypobaric chamber after a 10-h overnight fast to complete the trial under SL (757 ± 10 mmHg) or HA (459 ± 2 mmHg) conditions. To match our previous work (4), participants sat quietly while exposed to SL or HA conditions for 5 h. They then completed 80 min of steady-state exercise on a treadmill, while consuming 145 g of glucose (1.8 g/min) enriched with 200 mg [^13^C]glucose (Cambridge Isotope Laboratory, Andover, MA). Treadmill speed (3.7 ± 0.3 mph) and grade (2% ± 0%) were matched between conditions to match the absolute exercise intensity between SL (V̇o_2_: 1.66 ± 0.14 L/min, 329 ± 28 kcal) and HA (V̇o_2_: 1.59 ± 0.10 L/min, 320 ± 19 kcal). Glucose turnover was assessed using a primed (82.2 μmol/kg), continuous (0.78 μmol/kg/min) infusion of 6-6-[^2^H_2_]glucose, provided 2 h before and throughout exercise. Participants ingested 58 g carbohydrate immediately before exercise, followed by consumption of 29 g carbohydrate at 20, 40, and 60 min during exercise. The carbohydrate drink was prepared by the Combat Feeding Directorate (Natick, MA), containing corn-derived dextrose (CERELOSE, Ingredion, Westchester, IL). Nutrient content was confirmed using gas chromatography (Covance Laboratories, Inc., Madison, WI). Indirect calorimetry and breath sampling for ^13^C/^12^C expired in CO_2_ were used to determine carbohydrate and fat oxidation during exercise at SL and HA.

### Exogenous Glucose Oxidation and Turnover

As previously reported (1), exogenous glucose oxidation during metabolically matched, steady-state aerobic exercise was calculated using equations by Peronnet et al. (17) under SL and HA conditions. The Steele equation (18) with modifications for nonsteady state was used to calculate glucose *R*_d_ and MCR under SL and HA conditions. The differences in exogenous glucose oxidation, *R*_d_, and MCR between SL and HA conditions were used in the current analysis to determine associations of these primary outcome measures of the parent study to metabolite concentrations during exercise.

### Metabolomics

Serum samples for metabolomics analysis were collected by antecubital intravenous draw at 20 min before exercise under resting, fasted at SL, and after 40 min of aerobic exercise while consuming carbohydrate at SL and HA conditions. Samples were centrifuged at 3,000 rpm at 4°C for 10 min. Serum was then stored at −80°C until analysis. Samples were analyzed using four separate methods: two separate reverse phase (RP)/ultrahigh performance liquid chromatography-tandem mass spectroscopy (UPLC-MS/MS) methods with positive ion mode electrospray ionization (ESI), a RP/UPLC-MS/MS method with negative ion mode ESI, and a hydrophilic interaction (HILIC)/UPLCMS/MS method with negative ion mode ESI (Metabolon Inc., Morrisville, NC). Technical replicates, blanks, internal standards, and several recovery standards were analyzed with experimental samples for quality control. Raw data were extracted, peaks identified, and quality control processed using proprietary hardware and software. The relative quantitation values are based on integrated peak areas (area under the curve). All samples were analyzed on an equivalency basis based on volume.

Metabolites were identified by automated comparison of the ion features in the experimental samples to a references library of chemical standard entries that included retention time, molecular weight (*m*/*z*), preferred adducts, and in-source fragments as well as associated MS spectra, and were curated by visual inspection for quality control using software developed at Metabolon (Metabolon, Inc.) (19, 20). The level of identification for the majority of the compounds detected meets the highest standard of metabolite identification according to the Metabolomics Standards Initiative (21). Several types of controls were analyzed in concert with the experimental samples. A pooled matrix sample was generated by taking a small volume of each experimental sample to serve as a technical replicate throughout the dataset. Extracted water samples served as process blanks. A cocktail of quality control standards that would interfere with the measurement of endogenous compounds was spiked into every analyzed sample to allow instrument performance monitoring and aided chromatographic alignment. Instrument variability was determined by calculating the median relative standard deviation (RSD) for the standards that were added to each sample before injection into the mass spectrometers. Overall process variability was determined by calculating the median RSD for all endogenous metabolites (i.e., noninstrument standards) present in 100% of the pooled matrix samples.

### Statistical Analysis

Sample size calculations were based on primary study outcome of exogenous glucose oxidation, which has been previously reported (1). Analyses were completed using R v4.0.3, SPSS v26 (IBM Analytics; Armonk, NY), ArrayStudio (Omicsoft, Corp.; Cary, NC), and MetaboAnalyst v.5.0 (22). Before analysis of metabolomics data, any missing values were imputed using the minimum observed peak area for each compound. Peak area for each metabolite was then normalized to set the mean equal to 0, and log10 transformed to meet model assumptions. Metabolites were analyzed as change (Δ), calculated by subtracting peak area during postprandial/exercised state SL (ΔSL) and HA (ΔHA) from peak area during fasted, rested conditions at SL.

Orthogonal projections to latent structures discriminant analysis, hierarchical clustering of Euclidean distances, and pattern hunter analysis were conducted using MetaboAnalyst v.5.0 (22) to assess the effect of condition (ΔSL vs. ΔHA) on changes in global metabolite profiles. Paired *t* test was also used to determine differences between ΔSL and ΔHA. Statistically significant differences were then associated with between-condition differences (Δ) in exogenous glucose oxidation, glucose *R*_d_, and MCR using Spearman’s correlation coefficient. To account for multiple comparisons, the Benjamini–Hochberg method was used to estimate false discovery rate (*Q* value). Statistical significance was set at *P* < 0.05 and *Q *<* *0.10.

## RESULTS

As previously reported (1), exogenous glucose oxidation was 0.09 ± 0.09 g/min lower (*P* < 0.05), Glucose *R*_d_ was 1.66 ± 1.69 mg/kg/min lower, and MCR was 3.11 ± 3.00 mg/kg/min lower (*P* < 0.05) during metabolically matched, steady-state exercise at HA compared with SL.

**Figure 1.**
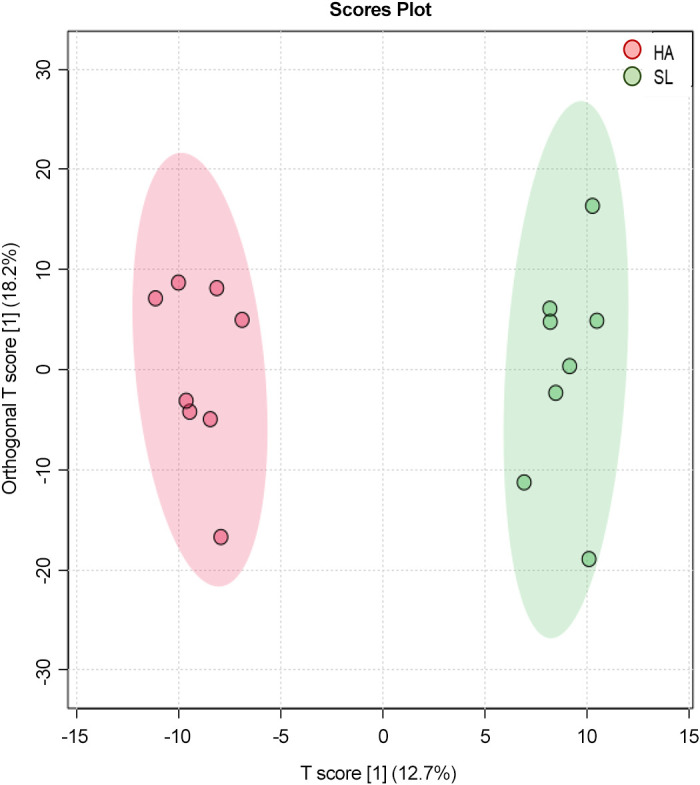
Orthogonal projections to latent structures discriminant analysis score plot for all metabolite features samples based on subject (*n* = 8) and condition [sea level (SL) and high altitude (HA)]. Circles represent individuals participants under each experimental condition.

**Figure 2.**
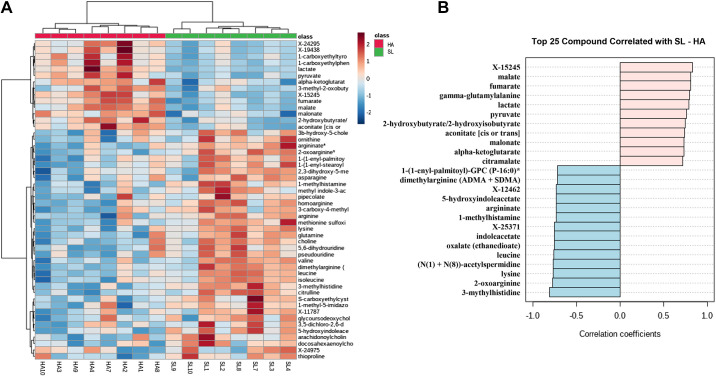
*A*: heatmap of hierarchical cluster analysis of the 50 metabolites with the lowest *Q* values between changes in sea level (ΔSL) and high altitude (ΔHA). *B*: pathfinder analysis of the 25 metabolites most highly associated with differences between ΔSL and ΔHA.

Metabolomics analysis measured 1,162 metabolites. Of those, 952 could be identified. Orthogonal projections to latent structures discriminant analysis demonstrated a clear separation in metabolite profiles by condition (Fig. 1). Hierarchical clustering and pattern search analysis identified multiple metabolites that clustered together and were most strongly associated with differences between conditions (Fig. 2, *A* and *B*). In total, 188 metabolites demonstrated significant (*P* < 0.05, *Q *<* *0.10) differences between ΔSL and ΔHA (Supplemental Table S1; see https://doi.org/10.6084/m9.figshare.14597352). Of those significant metabolites, 8% were within glycolysis and tricarboxylic acid (TCA) cycle pathways, 39% were within amino acid metabolism pathways, 2% were oxidative stress pathways, and 15% were within fatty acid metabolism pathways.

**Figure 3.**
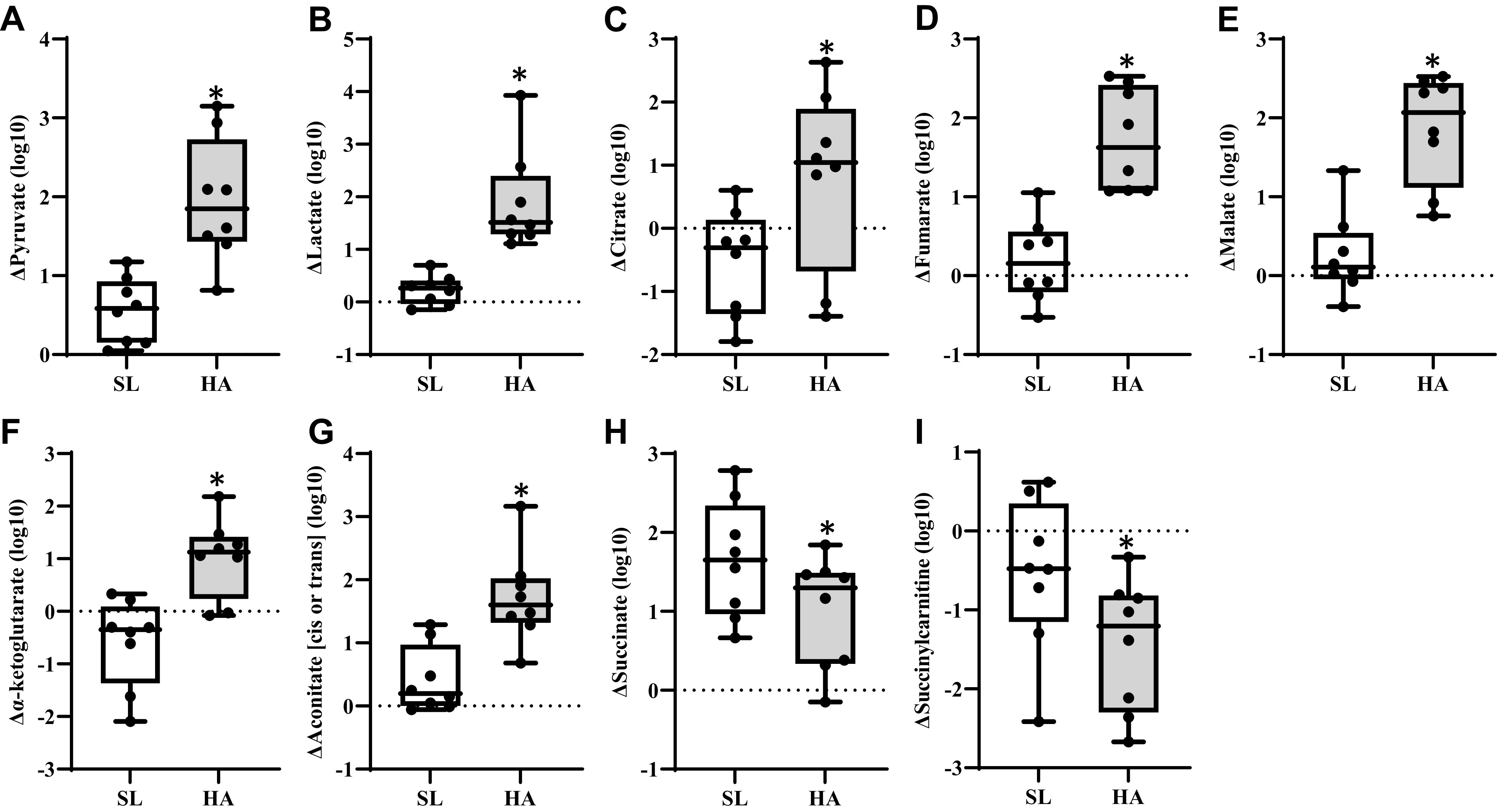
Box plots of difference from resting, fasted conditions at sea level (SL) in glycolysis (*A* and *B*), and tricarboxylic acid (TCA) metabolites (*C*–*I*) during exercise at SL and high altitude (HA). *Significantly different from SL, *P* < 0.05, *Q *<* *0.10. Exact *P* and *Q* values are reported in Supplemental Table S1.

Within the glycolysis pathway, increases in lactate and pyruvate were higher in ΔHA compared with ΔSL (Fig. 3, *A* and *B*). Increases in the TCA cycle metabolites malate, fumarate, citrate, aconitate (*cis* or *trans*), and α-ketoglutarate were also higher (*P* < 0.05, *Q *<* *0.10) for ΔHA compared with ΔSL (Fig. 3, *C*–*G*). Conversely, increases in succinate were lower (*P* < 0.05, *Q *<* *0.10) for ΔHA compared with ΔSL, and larger decreases (*P* < 0.05, *Q *<* *0.10) in succinylcarnitine were observed for ΔHA compared with ΔSL (Fig. 3, *H* and *I*).

**Figure 4.**
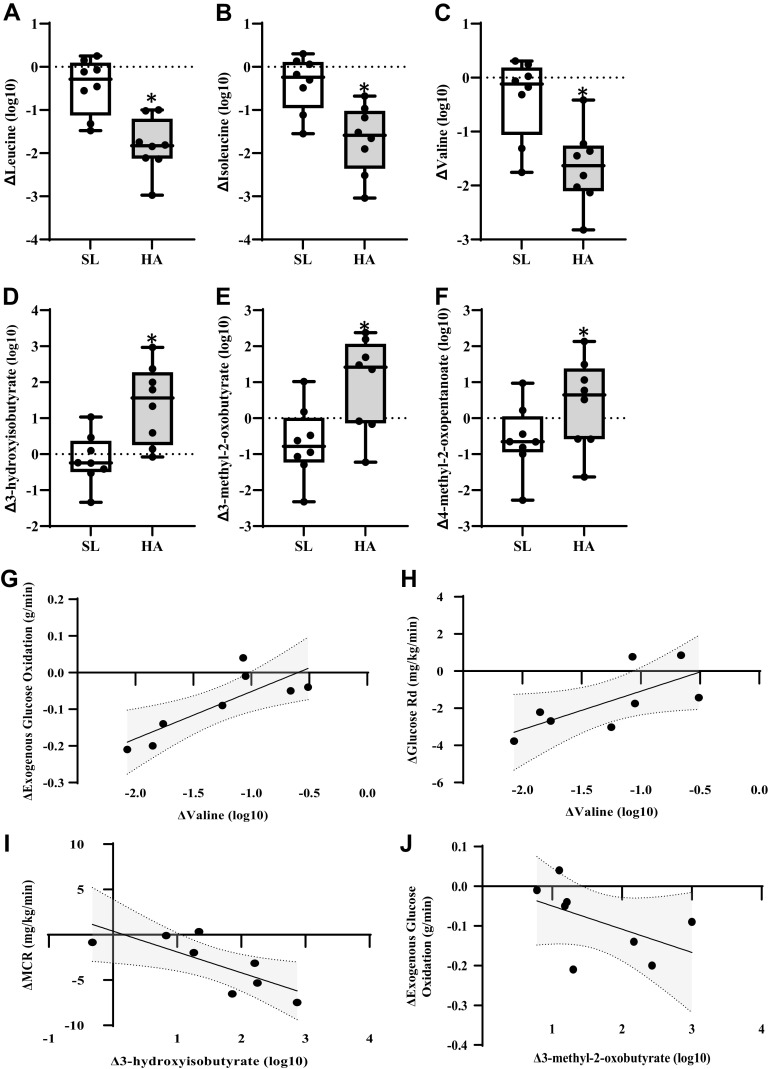
Box plots of difference from resting, fasted conditions at sea level (SL) in branched-chain amino acids (BCAA) metabolites (*A*–*F*) during exercise at SL and high altitude (HA). *Significantly different from SL, *P* < 0.05, *Q *<* *0.10. Exact *P* and *Q* values are reported in Supplemental Table S1. Significant associations in delta (HA-SL) BCAA metabolites to delta exogenous glucose oxidation, metabolic clearance rate (MCR), and glucose *R*_d_ (*G*–*J*).

Decreases in branched-chain amino acids (BCAA), leucine, isoleucine, and valine, were greater (*P* < 0.05, *Q *<* *0.10) for ΔHA compared with ΔSL (Fig. 4, *A*–*C*). Downstream BCAA metabolites, 3-hydroxyisobutyrate, 3-methyl-2-oxobutyrate, and 4-methyl-2-oxopentanoate, were higher (*P* < 0.05, *Q *<* *0.10) for ΔHA compared with ΔSL (Fig. 4, *D*–*F*). ΔValine was positively associated with Δexogenous glucose oxidation (*P* < 0.05, *r *=* *0.786) and Δglucose *R*_d_ (*P* < 0.05, *r *=* *0.786; Fig. 4, *G* and *H*). Δ3-Hydroxyisobutyrate and Δ3-methyl-2-oxobutyrate were inversely associated with ΔMCR (*P* < 0.05, *r* = −0.762) and Δexogenous glucose oxidation (*P* < 0.05, *r* = −0.738), respectively (Fig. 4, *I* and *J*). Within other pathways of amino acid metabolism, larger reductions in multiple histidine and urea cycle-related metabolites were observed for ΔHA compared with ΔSL (Table 1).

**Table 1.** Histidine and urea cycle metabolites

Subpathway	Metabolite	ΔSea Level	ΔHigh Altitude	*P* Value	*Q* Value
Histidine metabolism	1-Methyl-5-imidazoleacetate	0.30 (−0.20, 0.79)	−0.76 (−1.55, 0.03)	0.001965	0.034
	1-Methyl-5-imidazolelactate	0.23 (−0.27, 0.73)	−1.00 (−1.96, −0.04)	0.004776	0.048741
	1-Methylhistamine	0.62 (0.09, 1.16)	−0.68 (−1.23, −0.13)	0.000161	0.01762
	3-Methylhistidine	−0.52 (−0.95, −0.09)	−1.81 (−2.22, −1.40)	2.40 *E*−05	0.010267
	Histidine	−0.56 (−0.81, −0.31)	−1.56 (−2.36, −0.76)	0.009504	0.07169
	*N*-acetylhistidine	0.13 (−0.35, 0.61)	−0.74 (−1.34, −0.15)	0.004244	0.044842
Urea cycle metabolism	2-Oxoarginine	0.28 (−0.22, 0.77)	−0.97 (−1.41, −0.54)	0.001858	0.03393
	Argininate	0.07 (−0.43, 0.56)	−1.16 (−1.70, −0.63)	0.000257	0.01762
	Arginine	−0.72 (−1.16, −0.29)	−1.87 (−2.55, −1.19)	0.001006	0.025854
	Citrulline	−0.90 (−1.50, −0.29)	−2.06 (−2.47, −1.64)	0.000376	0.01762
	Dimethylarginine (ADMA + SDMA)	−0.18 (−0.98, 0.62)	−1.75 (−2.27, −1.23)	0.001237	0.026432
	Homoarginine	−0.41 (−1.10, 0.28)	−1.79 (−2.42, −1.16)	0.000442	0.01762
	*N*-acetylarginine	−0.36 (−1.23, 0.52)	−1.20 (−1.93, −0.48)	0.006469	0.056118
	Ornithine	−0.43 (−1.31, 0.44)	−1.47 (−2.22, −0.72)	0.001856	0.03393

Values mean (95% confidence interval) log10, presented as the delta during postprandial/exercise under sea level and high-altitude conditions minus fasted, rested sea level conditions. Main effect of condition for all metabolites; *P* < 0.05 and *Q *<* *0.10.

**Figure 5.**
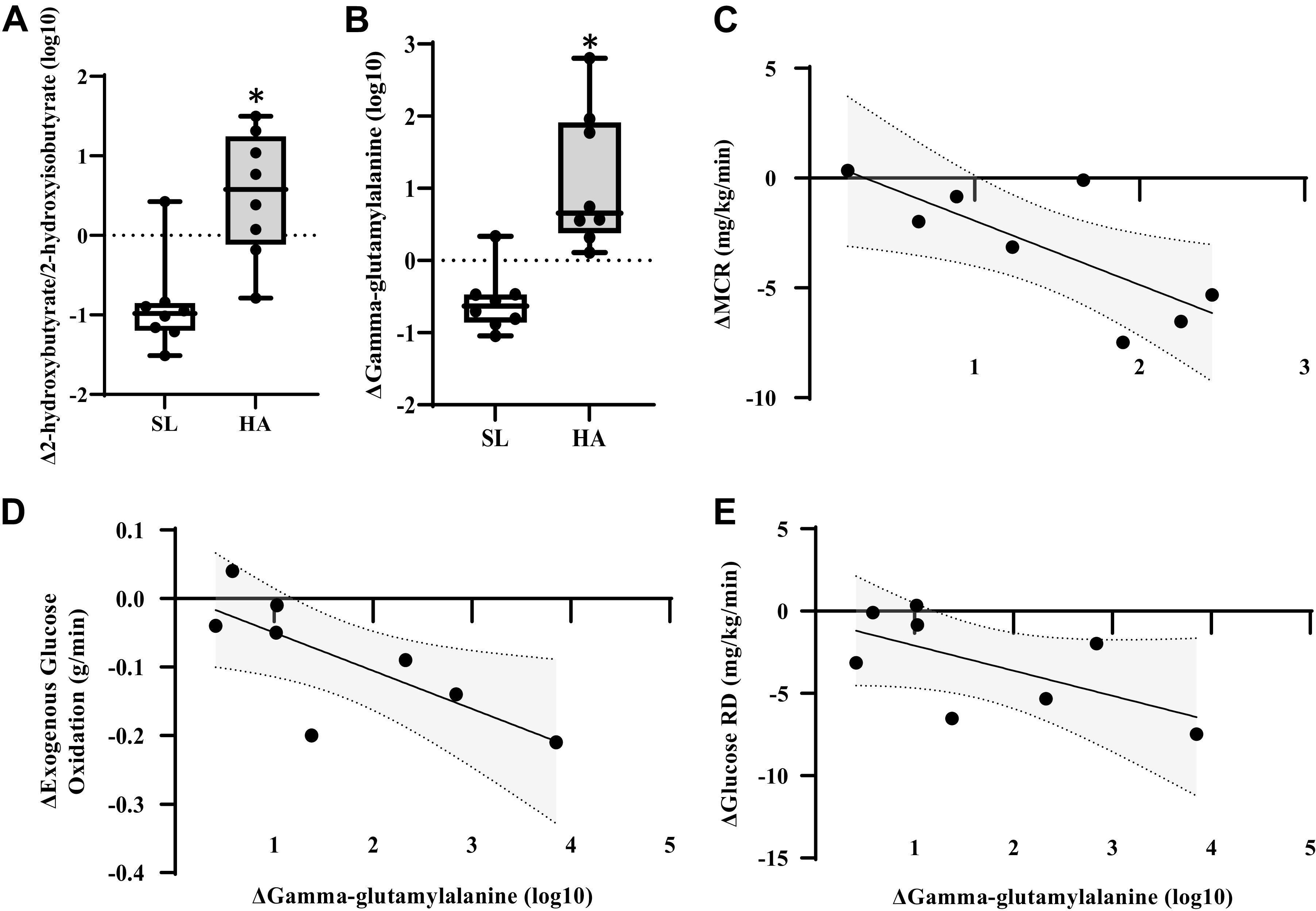
Box plots of difference from resting, fasted conditions at sea level (SL) in oxidative stress metabolites (*A* and *B*) during exercise at SL and high altitude (HA). *Significantly different from SL, *P* < 0.05, *Q *<* *0.10. Exact *P* and *Q* values are reported in Supplemental Table S1. Significant associations in delta (HA-SL) oxidative stress metabolites to delta exogenous glucose oxidation, metabolic clearance rate (MCR), and glucose *R*_d_ (*C*–*E*).

Changes in γ-glutamylalanine and 2-hydroxybutyrate/2-hydroxyisobutyrate, both markers of oxidative stress, were higher (*P* < 0.05, *Q *<* *0.10) for ΔHA compared with ΔSL (Fig. 5, *A* and *B*). Δ2-Hydroxybutyrate/2-hydroxyisobutyrate was inversely associated with ΔMCR (*P* < 0.05, *r* = −0.738; Fig. 5*C*). Δγ-Glutamylalanine was inversely associated with Δexogenous glucose oxidation (*P* < 0.05, *r* = −0.810) and Δglucose *R*_d_ (*P* < 0.05, *r* = −0.881; Fig. 5, *D* and *E*).

Changes in several fatty acid-related metabolites were different (*P* < 0.05, *Q *<* *0.10) between ΔHA compared with ΔSL (Table 2). Decreases in multiple carnitine and choline metabolites were greater (*P* < 0.05, *Q *<* *0.10) for ΔHA compared with ΔSL. In contrast, increase in malonate, a metabolite in the fatty acid synthesis pathway, was higher (*P* < 0.05, *Q *<* *0.10) for ΔHA compared with ΔSL.

**Table 2.** Fatty acid metabolites

Subpathway	Metabolite	ΔSea Level	ΔHigh Altitude	*P* Value	*Q* Value
Carnitine Metabolism	Carnitine	−0.19 (−0.74, 0.35)	−1.06 (−1.89, 0.23)	0.007039	0.058157
	Deoxycarnitine	−0.16 (−1.01, 0.69)	−0.76 (−1.55, 0.03)	0.006051	0.053835
Fatty acid metabolism (acyl carnitine, hydroxy)	(*S*)-3-Hydroxybutyrylcarnitine	0.34 (−0.17, 0.84)	1.45 (0.54, 2.37)	0.016166	0.096671
Fatty acid metabolism (acyl choline)	Arachidonoylcholine	−1.21 (−1.67, −0.74)	−1.97 (−2.43, −1.52)	0.000187	0.01762
	Docosahexaenoylcholine	−1.07 (−1.56, −0.57)	−1.85 (−2.28, −1.41)	0.000287	0.01762
	Eicosapentaenoylcholine	−1.09 (−1.69, −0.50)	−1.65 (−2.37, −0.92)	0.002779	0.036451
Fatty acid synthesis	Malonate	−0.91 (−1.81, −0.01)	1.01 (0.35, 1.67)	4.47 *E*−05	0.010267
Fatty acid, dicarboxylate	3-Carboxy-4-methyl-5-pentyl-2-furanpropionate (3-CMPFP)	−0.16 (−0.74, 0.43)	−1.46 (−2.22, −0.70)	0.002219	0.035351
	Octadecanedioate (C18)	−0.28 (−1.00, 0.44)	0.51 (−0.24, 1.27)	0.010877	0.076246
Phospholipid metabolism	Choline	−0.22 (−0.96, 0.51)	−1.63 (−2.49, −0.77)	0.002523	0.035729

Values mean (95% confidence interval) log10, presented as the delta during postprandial/exercise under sea level and high-altitude conditions minus fasted, rested sea level conditions. Main effect of condition for all metabolites; *P* < 0.05 and *Q *<* *0.10.

## DISCUSSION

The main finding of this study was that acute HA exposure altered circulating metabolomics profiles during metabolically matched, steady-state aerobic exercise compared with SL conditions. Significant differences were observed in metabolites within sub-pathways of glycolysis, TCA cycle, BCAA metabolism, oxidative stress, and fatty acid metabolism. Changes in several of these metabolites were associated with decreases in exogenous glucose oxidation, glucose *R*_d_, and MCR at HA, suggesting that changes in activity within these metabolic pathways under acute HA conditions may mediate hypoxia-induced insulin resistance.

The primary outcome from the parent study (1) was that exogenous glucose oxidation was reduced during aerobic exercise under acute HA conditions compared with SL. Accompanying lower rates of exogenous glucose oxidation were increased circulating glucose and insulin concentrations and reductions in glucose *R*_d_ and MCR (1). These shifts in glucose kinetics are characteristic of insulin resistance (7) and suggest that reductions in peripheral glucose uptake may be a primary factor contributing to lower rates of exogenous glucose oxidation at HA. We thus hypothesized that the observed dysregulations in glucose metabolism were the result of hypoxia-induced insulin resistance. Using nontargeted metabolomics analysis in this secondary investigation demonstrated greater increases in metabolites indicative of oxidative stress, γ-glutamylalanine, and 2-hydroxybutyrate/2-hydroxyisobutyrate, during exercise at HA relative to SL, which were associated with larger declines in exogenous glucose oxidation, MCR, and glucose *R*_d_. Increased oxidative stress is common during unacclimitized high-altitude exposure, potentially due to decreased oxygen pressure resulting in elevated free radical production and reductions in plasma antioxidant capacity (23, 24). Higher concentrations of these metabolites have also been observed in individuals with type 2 diabetes and insulin resistance (25, 26).

Increased oxidative stress in the current study may be associated with hypoxia-induced lipid accumulation under HA compared with SL conditions (27, 28). Lower concentrations of choline and acyl choline metabolites, carnitine, and higher concentrations of malonate under HA conditions indicate decreased fatty acid mobilization and fatty acid transport into the mitochondria, and increased fatty acid synthesis (29, 30). Low choline availability impairs fatty acid β-oxidation and results in mitochondrial dysfunction increasing oxidative stress (28, 31). Low carnitine and high acyl carnitine concentrations also increase oxidative stress and have been linked to insulin resistant populations (32, 33). Accumulation of acyl carnitines with hypoxia exposure has been reported in human (34) and rodent models (35), with the latter also reporting concurrent reductions in fat oxidation. Declines in the rate fat oxidation with HA exposure were reported in our parent study (1) and may have been the result of hypoxia-induced reductions in carnitine, which is essential for transporting long-chain fatty acids into the mitochondria for β-oxidation. Interestingly, supplementation of l-carnitine during hypoxia exposure functions as an antioxidant, reduced oxidative stress, and improved physical performance in rats (36, 37). Whether l-carnitine supplementation with acute HA exposure in humans can reduce oxidative stress to mitigate hypoxia-induced insulin resistance, and improve exercise performance is unclear.

Several BCAA metabolites that had a greater increase for ΔHA compared with ΔSL have also been previously associated with type 2 diabetes and insulin resistance (12). Higher concentrations of the BCAA metabolites 3-hydroxyisobutyrate, 3-methyl-2-oxobutyrate, and 4-methyl-2-oxopentanoate are associated with insulin resistance and type 2 diabetes in both human and mouse models (13, 38–40). In agreement with previous animal and human diseased state studies, our results demonstrate that greater increases in 3-hydroxyisobutyrate and 3-methyl-2-oxobutyrate were associated with lower MCR, an indicator of glucose uptake into peripheral tissue, and exogenous glucose oxidation, respectively.

Hypoxia-mediated changes in BCAA metabolites may, in part, be due to increased glycogenolysis and reductions in endogenous glucose stores during acute HA exposure. Our laboratory (1) and others (41–43) have shown endogenous glucose oxidation is higher and fat oxidation is lower during metabolically matched aerobic exercise under acute HA compared with SL. Increased glycogenolysis was reflected in the current study with higher increases in glycolysis metabolites pyruvate and lactate at HA. Higher pyruvate with HA exposure may explain the larger increase in TCA cycle metabolites during HA. However, concomitant increases in lactate production under the hypoxic conditions suggest increased anaerobic glycolysis. Higher pyruvate and lactate at HA may be due to increased hypoxia inducible factor 1α (HIF-1α) upregulating PDK1 via deactivation of PDH, preventing the conversion of pyruvate to acetyl-CoA (44). Increases in TCA cycle metabolites under HA conditions may instead reflect alternate carbon flow for oxidation (45). It is unlikely that increases in TCA metabolites are derived from fatty acids, as both fat oxidation and related fatty acid metabolites were reduced at HA. Greater decreases in leucine, isoleucine, and valine, with increases of downstream BCAA metabolites at HA may indicate increased reliance on BCAA carbon skeletons for energy production via the TCA cycle (46). Similar alterations in glycolysis, TCA, and BCAA metabolite profiles have been reported during ascent to Everest base camp (47) and following 4 days of exposure to hypobaric hypoxia at 5,300 m (34). Low energy or glycogen availability may increase the reliance of BCAA for substrate to be used for energy production and maintenance of glucose homeostasis (48, 49). Leucine and isoleucine can be broken down and converted to acetyl-CoA to enter the TCA cycle for energy production (46, 50). Acetyl-CoA then enters the TCA through its conversion to citrate, which aligns with higher concentrations of citrate at HA compared with SL in the current study.

Despite increases in the majority of TCA metabolites, succinate decreased to a greater extent during aerobic exercise at HA compared with SL. Lower succinate may be reflective of changes in BCAA metabolites. Unlike leucine and isoleucine, valine enters the TCA cycle through conversion to succinyl-CoA, which in turn is converted to succinate (46). Increase in 3-hydroxyisobutyrate, a valine metabolite, during exercise at HA may suggest impaired conversion of this metabolite to enter the TCA cycle to be used for energy. The subsequent higher increase in fumurate within the TCA cycle at HA may have resulted from the conversion of urea cycle metabolites for energy use (51). This increased reliance on urea and other amino acid metabolites for oxidative purposes may be a marker of negative net protein balance during acute hypoxia exposure. Our laboratory (52) has previously reported that reductions in plasma urea and histidine metabolites occur concurrently with negative net protein balance following 4 days of ∼55% activity-induced energy deficit. Reductions in concentrations of these metabolites have also been reported with decreased dietary protein intake (53), which results in negative protein balance. Taken together, these findings suggest that increased reliance on amino acids for oxidative purposes may, in part, help explain declines in mechanistic target of rapamycin complex 1 anabolic signaling (54), negative net protein balance (55, 56), and reductions in muscle mass (55–57) that occur in unacclimatized lowlanders sojourning at HA.

Our current study provides novel insight into the changes of metabolomics profiles while consuming carbohydrate during aerobic exercise under acute HA exposure. However, several limitations should be acknowledged. A sample size of eight for the parent study was generated based on anticipated differences of −14 ± 8 g/40 min exercise in exogenous glucose oxidation at HA compared with SL (1). This sample size was appropriate to address the primary outcome of differences in substrate oxidation and glucose turnover between SL and HA conditions; however, it is relatively small for metabolomics analysis. Though relatively small, other studies of similar sample size, *n* = 14 (7 men, 7 women) and *n* = 10 (6 men and 4 women) have effectively used metabolomics analysis to characterized metabolic alterations after 16 (10), 3, and 14 days (11) of hypoxia exposure. In addition, in the current study, the alterations in metabolomic profiles largely align with the results from the substrate oxidation in the parent study (1). Agreement between indirect calorimetry/isotope and metabolomic datasets, along with larger differences in effects between study conditions, enhances the physiological relevance and likely validity of the results. Furthermore, to account for the small sample size we assessed our data as deltas during exercise under acute high altitude and sea level conditions from our control resting/fasted sample collected under sea level conditions. This approach not only allowed us to isolate the effects of the exercise response between the two conditions, but also limited the number of data points being compared which enhance our statistical power. Similarly, the use of a crossover study designed enhanced the statistical power of our work by allowing each participant to act as their own control. It should also be noted that the results of our study should be taken in the context of our current study design. Our study exposed male participants to conditions equal to an altitude 4,300 m for ∼8 h. Only males were included in this investigation so outcome measures from the parent study could be appropriately compared with our previous work, which only had male participants. Sex-based differences in substrate oxidation during exercise at SL may result in differences in response to change in substrate oxidation at HA between men and women. Variant results may be observed using female participants, different severities of altitude, and more prolonged exposures.

It is important to state that while changes in metabolomics profiles during HA are reflective of changes in substrate oxidation and mirror profiles of insulin resistant populations, causality cannot be determined. The information obtained from this analysis can be used to identify potential interventions for future investigation to overcome changes in metabolite profiles to improve glucose tolerance to support physical performance at HA. Based on the present study findings, short-term use of insulin sensitizing drugs, such as metformin or pioglitazone, may be appropriate with unacclimatized HA exposure to efficiently metabolize dietary carbohydrate. Alternatively, potential nutrition interventions such as antioxidant supplement l-carnitine or choline supplementation to reduce oxidative stress and enhance fat oxidation may improve metabolic dysregulation by increasing reliance on fatty acids for fuel during exercise, minimizing oxidative stress associated with insulin resistance.

In conclusion, results from this study show differences in circulating metabolite profiles during exercise under acute HA compared with SL conditions, indicating increased glycolysis and TCA cycle activity, amino acid breakdown, oxidative stress, and fatty acid storage, and decreased fatty acid mobilization. Increased concentrations and inverse associations of metabolites within BCAA and oxidative stress pathways with exogenous glucose oxidation, glucose *R*_d_, and MCR suggest that changes in metabolite profiles under acute HA conditions may be reflective of hypoxia-induced insulin resistance. These data provide new insight into the potential underlying alterations in metabolic pathways that govern metabolic dysregulation in substrate oxidation under acute HA exposure.

## SUPPLEMENTAL DATA

Supplemental Table S1: doi.org/10.6084/m9.figshare.14597352.

## GRANTS

This material is based on the work supported by DHP JPC-5/MOMRP and through fellowship appointments to the US Army Research Institute of Environmental Medicine administered by the Oak Ridge Institute for Science and Education (to J. L. Coleman) through an interagency agreement between the US Department of Energy and the US Army Medical Research and Development Command.

## DISCLAIMERS

The opinions or assertions contained herein are the private views of the authors and are not to be construed as official or as reflecting the views of the Army or the Department of Defense. Any citations of commercial organizations and trade names in this report do not constitute an official Department of the Army endorsement of approval of the products or services of these organizations.

## DISCLOSURES

No conflicts of interest, financial or otherwise, are declared by the authors.

## AUTHOR CONTRIBUTIONS

L.M.M., A.A.F., A.J.Y., and S.M.P. conceived and designed research; L.M.M., M.A.W., A.J.Y., and S.M.P. performed experiments; L.M.M., J.P.K., and J.L.C. analyzed data; L.M.M., J.P.K., A.J.Y., and S.M.P. interpreted results of experiments; L.M.M. and J.L.C. prepared figures; L.M.M. drafted manuscript; L.M.M., J.P.K., M.A.W., J.L.C., A.A.F., A.J.Y., and S.M.P. edited and revised manuscript; L.M.M., J.P.K., M.A.W., J.L.C., A.A.F., A.J.Y., and S.M.P. approved final version of manuscript.
